# Soft Actor–Critic-Driven Adaptive Focusing under Obstacles

**DOI:** 10.3390/ma16041366

**Published:** 2023-02-06

**Authors:** Huan Lu, Rongrong Zhu, Chi Wang, Tianze Hua, Siqi Zhang, Tianhang Chen

**Affiliations:** 1 Interdisciplinary Center for Quantum Information, State Key Laboratory of Modern Optical Instrumentation, ZJU-Hangzhou Global Scientific and Technological Innovation Center, Zhejiang University, Hangzhou 310027, China; 2 School of Information and Electrical Engineering, Hangzhou City University, Hangzhou 310015, China; 3 China Aeronautical Establishment, Beijing 100029, China

**Keywords:** deep reinforcement learning, reconfigurable metasurface, focusing, soft actor–critic

## Abstract

Electromagnetic (EM) waves that bypass obstacles to achieve focus at arbitrary positions are of immense significance to communication and radar technologies. Small-sized and low-cost metasurfaces enable the accomplishment of this function. However, the magnitude-phase characteristics are challenging to analyze when there are obstacles between the metasurface and the EM wave. In this study, we creatively combined the deep reinforcement learning algorithm soft actor–critic (SAC) with a reconfigurable metasurface to construct an SAC-driven metasurface architecture that realizes focusing at any position under obstacles using real-time simulation data. The *agent* learns the optimal policy to achieve focus while interacting with a complex environment, and the framework proves to be effective even in complex scenes with multiple objects. Driven by real-time reinforcement learning, the knowledge learned from one environment can be flexibly transferred to another environment to maximize information utilization and save considerable iteration time. In the context of future 6G communications development, the proposed method may significantly reduce the path loss of users in an occluded state, thereby solving the open challenge of poor signal penetration. Our study may also inspire the implementation of other intelligent devices.

## 1. Introduction

Metasurfaces, as two-dimensional metamaterials [1,2], have attracted extensive attention owing to their ability to generate arbitrary EM arrays by introducing corresponding field discontinuities at interfaces. Several interesting EM devices are based on metasurface technology including couplers [3,4], cloaking [5,6,7], focusing or imaging systems [8,9,10,11], and other devices [12,13,14,15]. A focusing metasurface is one of the most thought-provoking devices and are of great significance in promoting research in fields such as radar detection, imaging, and 6G communications. In particular, the development of tunable metasurfaces/metamaterials in recent years has led to a significant increase in the freedom of designing reconfigurable functions [16,17,18,19,20,21,22,23,24,25,26,27]. For example, flexible technologies can achieve tunable focusing by mechanically controlling the expansion and contraction of a structure [17]. Similarly, other smart tunable materials such as varactor diodes [18], electrolyte elastomers [19], and phase-change films [20] can also achieve focusing effects at different positions and focal lengths using reasonable voltage or light modulation. However, these traditional techniques for achieving focus are direct calculations of the compensated phase, and the traditional theoretical calculations fail when there is an obstacle to the incident wave source and metasurface.

An ideal metasurface-focusing system should quickly realize focusing tasks in different environments to adapt to different communication scenarios. In particular, in the presence of unknown obstacles, the fast realization of the focusing task is of great importance for user signal transmission and reception. However, this is difficult to achieve because the amplitude-phase characteristics of the unit cell are difficult to analyze, resulting in the inability to analytically deduce the state of each meta-atom. Therefore, intelligent adaptive strategies are urgently required. Although adaptive optics have been extensively studied by enabling artificial intelligence (deep learning) on metasurfaces [28,29,30], the success of deep learning requires prior knowledge of the environment. Unlike traditional deep learning methods, which require a large number of training and test sets related to a specific environment [31,32,33], another branch of machine learning, reinforcement learning (RL), is used to describe and solve problems in which *agents* learn strategies to achieve specific goals in the process of interacting with the environment [34,35,36]. In particular, the development and improvement of deep RL (DRL) in the fields of Go and robotics has increased the demand for RL [37,38,39,40]. This makes it possible to solve focusing tasks in complex environments; however, it remains challenging.

In this study, we combine the DRL algorithm, soft actor–critic [41,42], with a metasurface to propose the SAC-M system for adaptive focusing design under an arbitrary obstacle. We first analyzed the adaptive focusing framework and implementation process of the SAC algorithm and used the designed metasurface to simulate and train the focusing task in the presence of different obstacles. Simulation results show that the proposed SAC-M system can operate stably in the presence of multiple obstacles of any shape and adaptively converge incident waves to user-defined locations. In addition, the knowledge learned by the *agent* based on the maximum entropy strategy in an environment can be used to initialize a new environment where the SAC-M system can learn a strong generalization ability. Both the simulation and network training results demonstrated that SAC-M exhibits effective and robust adaptive focusing capabilities when dealing with complex EM environments. Moreover, the SAC-M architecture is beneficial for the proposal of other EM wave smart devices and may be extended to other research fields, such as communication to solve more challenging problems.

## 2. Materials and Methods

### 2.1. Architecture of the SAC-M

**Figure 1:**
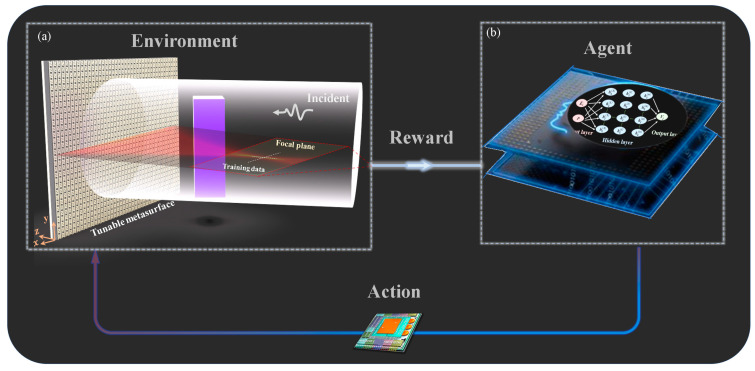
Proposed SAC-M Architecture. (**a**) Incident wave, metasurface, and object constitute complex environments simultaneously. (**b**) *Agent* continuously collects and analyzes data in real time.

The advantage of SAC-DRL lies in its ability to explore more ways to solve problems while learning the policy. For different focuses in unknown scenarios, the *agent* can rapidly complete new tasks and reduce unnecessary iterations such that it can be adapted to different types of environments. Figure 1 illustrates the proposed SAC-M mechanism. A plane incident wave, tunable metasurface, and arbitrarily shaped object (purple cube) simultaneously constitute a complex environment, as shown in Figure 1a. Two sets of one-dimensional data (crossed white-dotted lines) of the focal position in the focal plane (red dashed box in Figure 1a) were selected as the training data. When the state of the tunable metasurface changed, the state (training data) obtained at each moment changed accordingly. The agent collected the training data in the environment, compared it with the objective function (Figure 1b), and analyzed the contribution (termed rewards) of state variables to the final task in real time. Following the analysis, the output action initiated, which means that the voltage or capacitance change at the next moment is transmitted to the tunable metasurface, thereby altering the environment. The training data also changed in real time and were obtained by the agent. Finally, the SAC-M framework formed a closed-loop architecture of an environment-agent-action-environment and iterated continuously until the mission was accomplished.

### 2.2. SAC Algorithm

In traditional DRL, the *agent*’s goal is to learn a policy that maximizes the accumulated reward expectation [40]. It considers only one optimal action for a state and does not have the ability to cope with changing environments. However, the SAC-DRL is based on maximum entropy, and its core idea is to randomize the strategy and disperse the probability of each action output as much as possible, and not leave out any useful action and trajectory. The optimal strategy is defined as [41,42]:

(1) $$\pi^{*}=argmax_{\pi }E_{\left(s_{t},a_{t}\right)}[\sum_{t}R\left(s_{t}s_{t},a_{t}\right)+\alpha H(\pi (\cdot |s_{t}))]$$

where $\pi^{*}$ is the optimal strategy; $s_{t}$ and $a_{t}$ are the state and action at time t, respectively; $R\left(s_{t},a_{t}\right)$ represents the return; $H(\pi (\cdot |s_{t})$ is the entropy; and α is the temperature parameter, which determines the randomness of the strategy.

The neural network explores all possible optimal ways for the learned policy to have stronger exploration and robustness and can be used to initialize more complex tasks. In other words, when the policy has completed the focusing task in an environment, the *agent* can update the policy faster when faced with a new environment.

**Figure 2:**
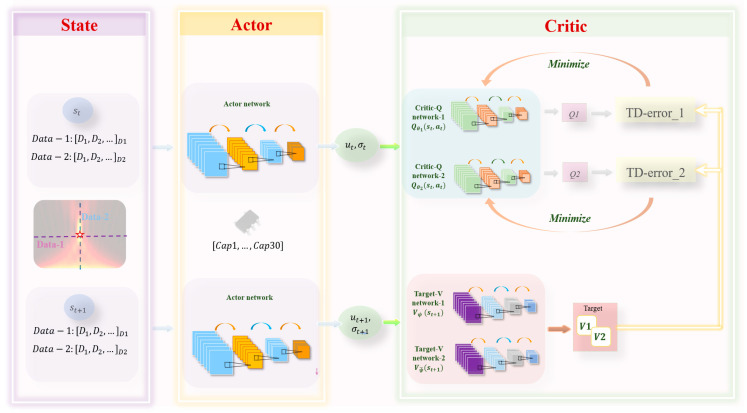
Architecture of SAC. State ($s_{t}$) is the data of the focal plane, and the action is the capacitance sequence: $\left[Cap1,\ldots ,Cap30\right]$. SAC network consists of one actor network and four critic networks (Q-network-1, Q-network-2, V-network-1, and V-network-2).

Figure 2 shows the network framework of the SAC, including the state, actor, and critic. We used four-layer convolutional networks to build the actor and critic networks. $S_{t}$ is the state (electric field) collected by the *agent* from the environment at time t. The actor network selects the appropriate action (capacitance matrix $\left[Cap1,\ldots ,Cap30\right]$) based on the state, and the critic network evaluates the value of the state. For continuous actions, the actor outputs the mean ($u_{t}$) and variance ($\sigma_{t}$) of the action distribution.

To stabilize the training, “critic” uses two Q-value functions represented by θ1 and θ2, and uses two value functions, represented by $\bar{\psi }$ and ψ. The Target-V network represents the estimation of the state value, and the Critical-Q-network represents the estimation of the action value. In the SAC algorithm, the goal of the actor is to maximize the output action using Equation (1). The goal of the Critic-Q and Target-V networks is to make the output action value Q and state value V more accurate.

Similar to the traditional actor–critic algorithm, the network update iteration of SAC is divided into two steps: soft-policy evaluation and soft-policy improvement. In the soft-policy evaluation, the policy is fixed, and the Q value is updated using the Bellman equation until convergence occurs.

(2) $$Q_{soft}^{\pi }\left(s_{t},a_{t}\right)=r\left(s_{t},a_{t}\right)+\gamma E_{s_{t+1,}a_{t+1}}[Q_{soft}^{\pi }\left(s_{t+1},a_{t+1}\right)-\alpha \log(\pi (a_{t+1}|s_{t+1})]$$

In soft-policy improvement, the policy is updated using Equation (3).

(3) $$\pi^{\prime }=\underset{\pi_{k}\in \Pi }{argmin}D_{KL}(\pi_{k}(\cdot |s_{t})||\frac{\exp(\frac{1}{\alpha }Q_{soft}^{\pi }\left(s_{t},\cdot \right))}{Z_{soft}^{\pi }\left(s_{t}\right)})$$

Soft-policy iteration algorithms alternate the soft-policy evaluation and soft-policy improvement steps. The detailed derivation process of the algorithm can be found in the references [41,42].

### 2.3. Element Configuration

**Figure 3:**
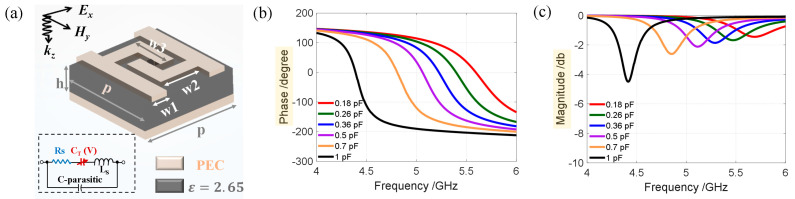
(**a**) 3D view of the designed unit cell. p=10 mm,w1=1.5 mm,w2=4.2 mm,w3=4.5 mm,h=2.5 mm. (**b**,**c**): Reflection phase and amplitude at different capacitances and frequencies, respectively.

The tunable reflective metasurface arrangement is composed of a group of unit cells with various capacitances. The physical dimensions of the proposed reconfigurable metasurface were $300\times 200{\;\mathrm{mm}}^{2}$, comprising 30×20 unit cells along the x and y directions, where y direction was set as the periodic boundary. Figure 3a shows that the designed unit cell, which consists of a $10\times 10{\;\mathrm{mm}}^{2}$ substrate ($\varepsilon_{r}=2.65$) with 2.5 mm thickness, a fully reflective metal patch attached to the back, and a specially designed metal structure with a varactor diode. The varactor diode model is MAVR-000120-14110P, whose capacitance can be tuned between 0.14 and 1.1 pF with a parasitic resistance of approximately 2.5 Ω. Figure 3a shows an equivalent circuit diagram. The RC model is used as an equivalent diode, and the diode characteristics can be changed by adjusting capacitance. The metal sheet attached to the front is a center-symmetrical figure with a diode placed at the center. Using the commercial software CST2020 (CST Studio Suite 2020, Dassault aircraft company, France) to analyze the S-parameters of the unit cell, the frequency domain simulation mode is used, its x and y directions are set as the unit cell boundary, and the electric field is along the x direction. The S-parameters are shown in Figure 3b,c. Apparently, at the frequency f=5 GHz, the reflection phase changes continuously to 320° and the amplitude is greater than −2 dB. Here, we show six kinds of capacitors as characteristic units, and the capacitance values are 0.18, 0.26, 0.36, 0.5, 0.7, and 1 pF.

## 3. Results and Discussion

### 3.1. The Training Results and Unit Cell Design

**Figure 4:**
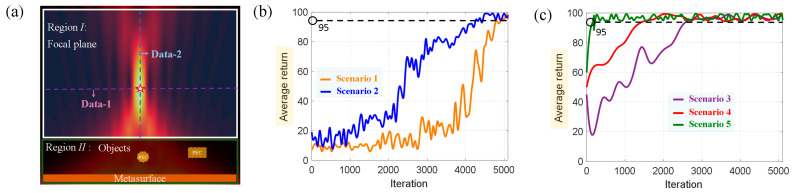
(**a**) Schematic illustration of focal plane and selection of training data. (**b**) Average returns for Scenarios 1 and 2. (**c**) Average returns for Scenarios 3, 4, and 5.

The selection of the state data is crucial for the *agent* to quickly learn a good policy. To reduce the amount of data storage, we only selected two sets of data (dotted line in Figure 4a) at the focal position (the five-pointed star) in the focal plane as the input of the *agent*, denoted as $Data-1:\;{\left[D_{1},\;D_{2},\;\ldots ,\;D_{75}\right]}_{D1}$; $Data-2:\;{\left[D_{1},\;D_{2},\;\ldots ,\;D_{36}\right]}_{D2}$. In Figure 4a, the solid white frame is the focal plane, and any focal position in this area can be defined as an *agent-learning* target. The green solid line frame represents the area where the object and metasurface are located. The object was a perfect electric conductor (PEC). Objects of any shape and number can be placed, and the *agent* does not need to be known in advance. Before the *agent* learns, we first define the target data: $Goal-1:\;{\left[D_{1},\;D_{2},\;\ldots ,\;D_{75}\right]}_{G1}$; $Goal-2:\;{\left[D_{1},\;D_{2},\;\ldots ,\;D_{36}\right]}_{G2}$, which were obtained by the traditional focusing method [43]. The purpose of the *agent* is to learn a policy making the Data−1 and Data−2 rapidly approach Goal−1 and Goal−2 in any scenario. Here, we defined five scenarios, as listed in Table 1.

Figure 4b,c show the curves of the average return of the *agent* in the learning process with the number of iterations under different scenarios. A good focusing effect is generated when the average returns to 95. When the *agent* faces a new scene for the first time, large amounts of data are necessary, as the *agent* knows nothing about the environment. As shown in Figure 4b, the *agent* obtains a good policy after approximately 5000 iterations for Scenario 1 (orange curve). In Scenario 2, the *agent* continues to use the experience and policy learning in Scenario 1 to keep testing the new environment, and finally achieves a good focusing effect after approximately 4000 iterations. For the new task in Scenario 2 (blue curve), the *agent* already has a certain learning ability compared with Scenario 1. At this time, we changed the scene again, as shown in Figure 4c, and the new focusing task could be achieved after 2000 iterations in Scenario 3 (purple curve). The amount of data was twice as small as before because the *agent* combined the policies of Scenarios 1 and 2, and the learning ability was further improved. Finally, we changed the environment to Scenarios 4 and 5. At this time, the speed of policy evaluation and policy improvement was faster. In particular, in Scenario 5, the *agent* only required a few dozen iterations to complete the focusing task. Combining the training data of different scenarios, it can be observed that the *agent* learned the solution to a set of problems and can quickly adapt to the new environment with the help of empirical knowledge.

### 3.2. Adaptive Focusing Results at Different Positions

**Figure 5:**
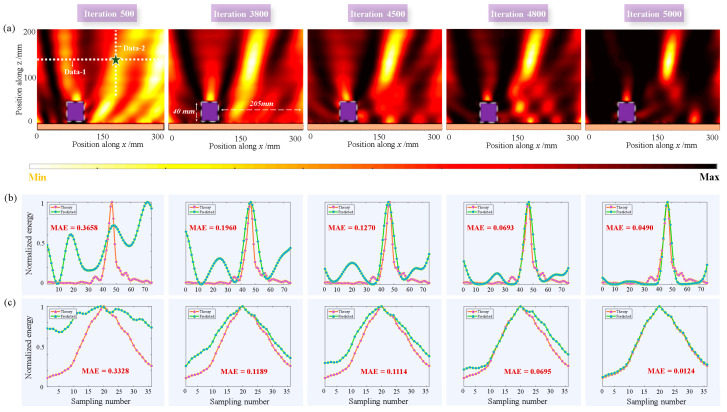
Focusing process in Scenario 1. (**a**) Dynamic focusing process with different iterations under normal incidence. Black five-pointed star represents the position of the target focus and the white-dotted line represents the data obtained by the *agent*. The length of the sides of the object was 40 mm. (**b**) Network prediction and theoretical results of Data 1 under different iterations and the MAE loss was calculated. (**c**) Network prediction and theoretical results for Data 2 under different iterations.

Figure 5 shows the focusing process in Scenario 1 under normal incidence. As shown in Figure 5a, the black five-pointed star marks the position of the target focus point, and the row and column data where the position is located are selected as training data (white-dotted line). The black square area represents an obstacle. In Scenario 1, an object with a side length of 40 mm was located 5 mm above the metasurface, and the object was 205mm away from the edge of the metasurface. The five images in Figure 5a show the electric field energies at iterations of 500, 3800, 4500, 4800, and 5000 times. Scenario 1 is the unknown environment faced by the *agent* for the first time; therefore, the process of learning the policy was very long. It can be observed that when the iteration was 3800 times, a certain focusing effect was produced, but the energy was relatively scattered at this time. When the iteration reached approximately 5000 times, the energy almost converged to the position of the target focus, and the *agent* completed the task in Scenario 1. The plot in Figure 5b,c show the predicted (green line) and theoretical (red line) values for Data 1 and Data 2, respectively, and we calculated the mean absolute error (MAE) loss for both. With the continuous learning of the *agent*, the predicted value constantly approached the theoretical value, and the MAE constantly approached zero. The MAE is defined as:

(4) $$MAE=\frac{1}{m}\sum_{i=1}^{m}\left|E_{Theory}-E_{Predict}\right|$$

where *m* is the total number of iterations, $E_{Theory}$ is the theoretical value, and $E_{Predict}$ is the network prediction. The closer the MAE was to 0, a better the training result was realized.

**Figure 6:**
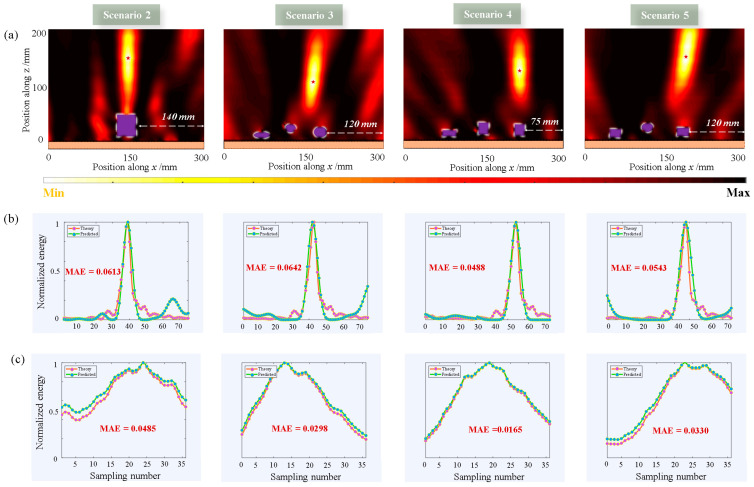
Focusing results for different scenarios. (**a**) Two-dimensional focusing results for different scenarios under normal incidence. Red five-point star represents the position of the target focus. (**b**) Network prediction and theoretical results of Data 1 under different scenarios, and the MAE loss was calculated. (**c**) Network prediction and theoretical results for Data 2 under different scenarios.

Figure 6 shows the focusing results for Scenarios 2, 3, 4, and 5. The shape and number of objects are different in different scenes, and the red star is the focus position. Combining previous experiences and strategies, the *agent* can rapidly adapt to the new environment and achieve efficient focusing at the target location, as shown in Figure 6a. Figure 6b,c show the theoretical (red line) and predicted (green line) curves of Data 1 and Data 2, respectively, under different scenarios. It can be observed that the two have excellent fitting effects.

## 4. Conclusions

In conclusion, combined with a soft actor–critic and reconfigurable metasurface, we proposed and designed an SAC-M-driven adaptive focusing system. The *agent* learns and improves policies in real time in changing environments, and the metasurface is guided by it and exhibits effective and robust adaptive focusing capabilities based on 1D electric-field data. The simulation and network results demonstrated that the proposed SAC-M system is highly adaptable for achieving focus at arbitrarily specified positions with multiple objects of any shape. Our novel combination of the classical EM theory and mainstream theories of RL uncovered the exciting potential of metasurfaces. The proposed SAC-M framework not only provided an idea for realizing adaptive focusing in unknown environments, but also provided a general architecture to solve more challenging problems. If similar designs can be decoded, smart metasurface systems offer incredible potential for communication and radar technologies.

## Figures and Tables

**Table 1:** Define of different scenarios.

Scenario	Focal Position (x, z)/mm	Object
Scenario 1	(188, 130)	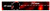
Scenario 2	(150, 130)	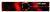
Scenario 3	(169, 100)	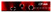
Scenario 4	(225, 110)	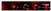
Scenario 5	(195, 150)	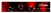

## Data Availability

The data are available from the author.
